# Young Symptomatic Female Patient With IgG4-Related Benign Biliary Stricture: A Case Report

**DOI:** 10.7759/cureus.86833

**Published:** 2025-06-26

**Authors:** Faisal Janahi, Khalifa Yusuf, Mohamed Alchalban, Sagar Adkar

**Affiliations:** 1 Internal Medicine, Bahrain Defence Force Hospital, Riffa, BHR; 2 Radiology, Bahrain Defence Force Hospital, Riffa, BHR

**Keywords:** biliary diseases, chronic abdominal pain, diagnostic and therapeutic ercp, igg4 disease, sclerosing cholangitis

## Abstract

Biliary strictures occur due to the narrowing of the bile ducts, leading to impaired normal flow of bile. This leads to a spectrum of symptoms ranging from being asymptomatic to displaying symptoms secondary to obstruction. Hence, we present a case of a young female patient presenting to the Emergency Department with recurrent abdominal pain requiring intravenous analgesia; imaging shows stricture of the common bile duct with no pathological lesions but elevated plasma IgG4 levels. Our case is particularly unique because the patient displays no systemic features of IgG4-related disease yet requires serial endoscopic retrograde cholangiopancreatography (ERCP) and oral corticosteroids. Given the peculiarity of such presentation in a young female patient, this case contributes to the ongoing research on the heterogeneity of IgG4-related diseases.

## Introduction

Biliary strictures occur due to the narrowing of the intrahepatic or extrahepatic ductal segment. The occurrence of those strictures hinders the normal flow of bile, resulting in the clinical picture of biliary obstruction. Biliary strictures can be further categorized as being congenital or acquired. Acquired biliary strictures can be either benign or malignant. The incidence of biliary stricture varies based on the aetiology [1,2]. The clinical presentation is often variable; some patients may be picked up on routine imaging and are asymptomatic, and some may present with obstructive symptoms such as jaundice and pruritus.

Laboratory investigations of biliary stricture may show a cholestatic pattern of liver injury with elevation in alkaline phosphatase, but in some cases, liver enzymes can be unremarkable [2]. The clinical and biochemical presentation of biliary strictures makes the diagnosis challenging for clinicians. This article describes a case of biliary stricture presenting with recurrent abdominal pain necessitating intravenous analgesia with elevated plasma IgG4, endoscopic retrograde cholangiography with stent placement, and oral prednisolone.

## Case presentation

A 21-year-old female patient first presented to the Emergency Department in January 2021 with a complaint of epigastric pain of two days in duration, radiating to the back. She had been suffering from intermittent pain suggestive of biliary colic for one year, but it had gotten worse lately. There was no past or family history of tuberculosis and no significant past surgical history. She is known to have epilepsy on Keppra 750 mg orally twice daily.

Her laboratory studies were remarkable for the elevation of liver enzymes with normal amylase (Table 1).

**Table 1:** Laboratory investigations including liver enzymes and serum amylase Normal reference ranges are provided in the first column within parentheses

Laboratory investigations	January 2021	June 2021	December 2021
Total bilirubin (0.0–24.0 μmol/L)	10.9	12.7	8.5
Alkaline phosphatase (ALP) (40.0–129.0 IU/L)	164.0	109.0	89.0
Alanine aminotransferase (ALT) (0.0–41.0 IU/L)	195.6	73.4	12.7
G-glutamyl transferase (GGT) (8.0–61.0 IU/L)	474.0	133.0	27.0
Aspartate aminotransferase (AST) (0.0–40.0 IU/L)	391.1	72.5	19.8
Amylase (28.0-100.0 IU/L)	44.0	65.0	62.0

**Figure 1:**
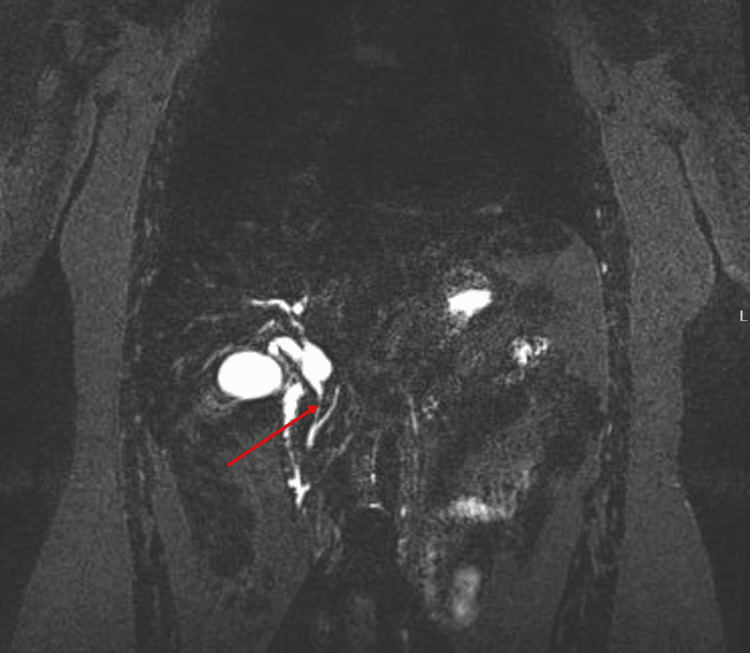
Magnetic resonance cholangiopancreatography (MRCP) T2-weighted image showing common bile duct stricture (red arrow)

Ultrasound of the abdomen showed common bile duct dilation with sludge, and polycystic ovaries were noted. Magnetic resonance cholangiopancreatography (MRCP) showed calculous gallbladder disease, a few small stones in the cystic duct, and a common hepatic duct with long stricture of the common bile duct (Figure 1).

**Figure 2:**
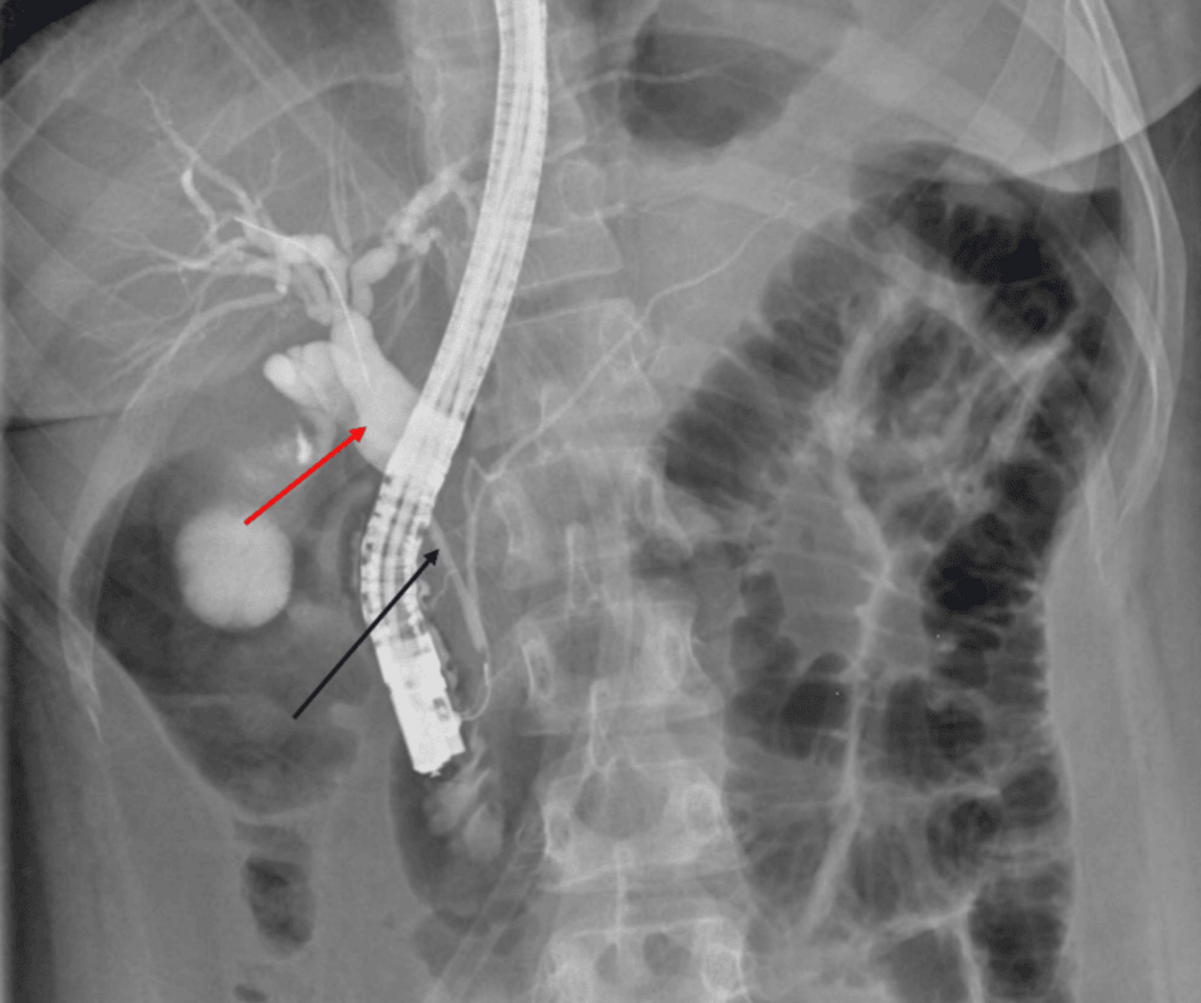
Endoscopic retrograde cholangiopancreatography (ERCP) showing narrow common bile duct (dark blue arrow) and dilated common hepatic duct (red arrow) with transition point behind the scope

**Figure 3:**
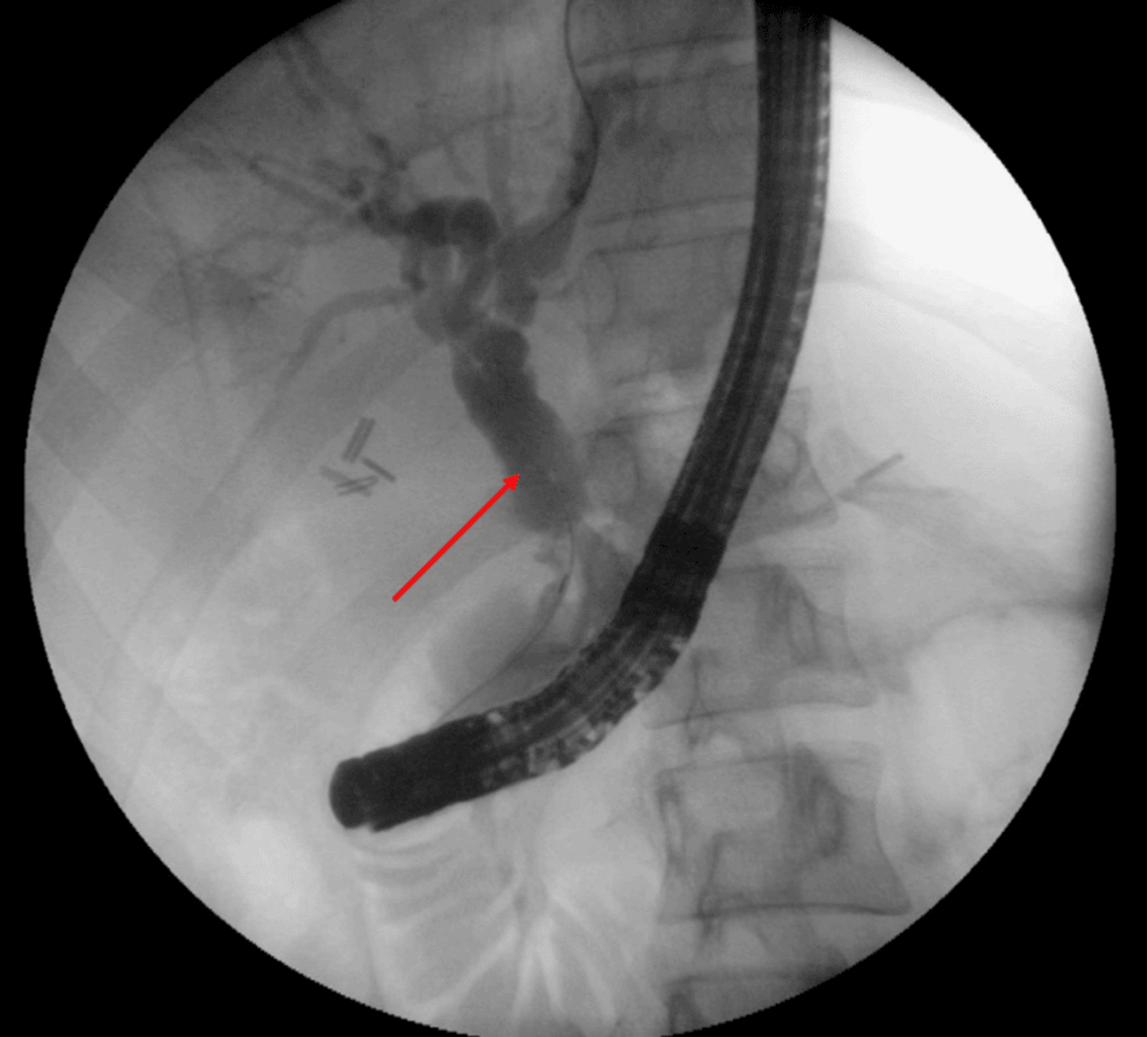
Endoscopic retrograde cholangiopancreatography (ERCP) showing dilated common hepatic duct (red arrow)

Endoscopic retrograde cholangiopancreatography (ERCP) (Figures 2, 3) was done, which showed dilated intrahepatic biliary radicals (IHBR) with common hepatic duct (CHD) and sharp shouldering at the level of cystic duct insertion. The common bile duct (CBD) was narrow; a few stone fragments were extracted with balloon sweeps, and a plastic biliary stent was placed with brush sampling from the lower CBD. Biliary sampling was negative for malignancy. Contrast-enhanced computed tomography (CT) abdomen confirmed the CBD stricture and excluded hepatopancreatobiliary malignancy. The pancreas was normal.

Work-up for biliary stricture revealed elevated serum IgG4 1.860 g/L. Antineutrophil cytoplasmic antibody (ANCA) and TB quantiferon were negative. Tumor markers were negative.

Two weeks later, she underwent laparoscopic cholecystectomy with pathology confirming acute on chronic cholecystitis.

She was kept on ursodeoxycholic acid and a proton pump inhibitor. She underwent elective ERCPs in March 2021 and June 2021, with two biliary stents placed with stricture dilatation. Biopsies were taken twice, from the ampulla and lower CBD, and revealed active chronic duodenitis with few positive IgG4 plasma cells. Repeated serum IgG4 level was also elevated at 1.857 g/L.

A gastroscopy was performed, which showed mild antral gastritis only.

**Figure 4:**
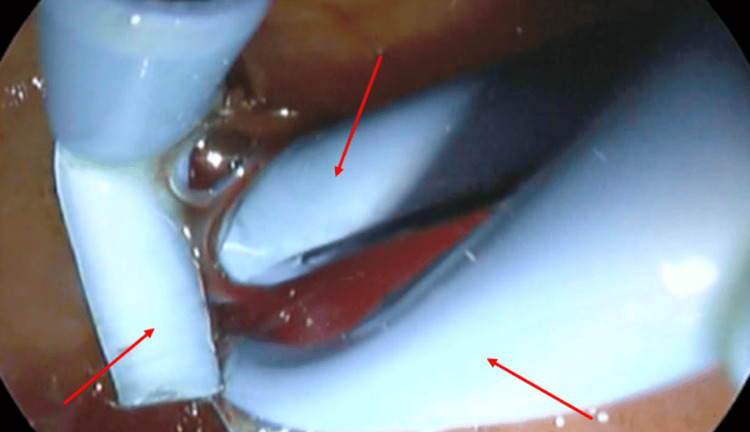
Endoscopic retrograde cholangiopancreatography (ERCP) showing placement of three biliary stents (red arrows)

The patient continued to have episodes of abdominal pain radiating to the back, requiring intravenous analgesics three to four times per month. She was started on steroids for IgG4-related benign biliary stricture. Her liver enzymes improved, with normal bilirubin and transaminases. She was advised to undergo further serial ERCP, three to four months apart, with the placement of an increasing number of biliary stents (Figure 4).

## Discussion

This case underscores the diagnostic dilemma of a young female patient presenting with recurrent abdominal pain suggestive of biliary colic and ultimately found to have common bile duct stricture on imaging with elevated serum IgG4 levels in the absence of the systemic manifestations associated with IgG4-related disease. The Japanese Biliary Association has established clinical diagnostic criteria for IgG4-related sclerosing cholangitis, which include biliary imaging findings of diffuse or segmental narrowing of bile ducts, elevation in serum IgG4 levels (135 mg/L or higher), coexistence of other IgG4-related diseases (e.g., autoimmune pancreatitis), and characteristic histopathological findings [3].

Magnetic resonance cholangiopancreatography (MRCP) revealed a common bile duct (CBD) stricture, which necessitated proceeding with endoscopic retrograde cholangiopancreatography (ERCP), which demonstrated the abnormality. While contrast-enhanced computed tomography (CT) of the abdomen did not reveal any mass or pancreatic lesions, the raised serum IgG4 increased the possibility of IgG4-related disease. The absence of other autoimmune conditions, such as autoimmune pancreatitis associated with IgG4-related disease, makes this case quite peculiar. Most published cases in the literature related to IgG4-related sclerosing cholangitis are associated with autoimmune pancreatitis type 1 [4]. However, in our patient, the absence of the typical systemic features and the age pose a diagnostic dilemma.

One of the diagnostic criteria of IgG4-related sclerosing cholangitis is elevation of serum IgG4 (135mg/L or higher). Our patient's serum IgG4 level was elevated during hospitalization and subsequent follow-ups despite serial endoscopic retrograde cholangiopancreatography (ERCP) and oral corticosteroid therapy. A study conducted by Tang et al. revealed that serum IgG4 levels might vary among different extents of organ involvement [5]. While elevated serum IgG4 levels decrease with corticosteroid therapy, similar effects can also be seen in conditions unrelated to IgG4-related disease [6]. Thus, it can be unreliable as a single marker to monitor disease activity.

In a study conducted by Naitoh et al., liver biopsies were performed to distinguish IgG4-sclerosing cholangitis from primary sclerosing cholangitis. It revealed the usefulness of liver biopsy in detecting small bile duct involvement in patients with intrahepatic biliary stricture picked up on cholangiography [7]. This study highlights the role of liver biopsy in cases that remain inconclusive or those with no apparent cause. In our present case, the patient had not undergone a liver biopsy but had undergone serial ERCP with dilatation for the biliary stricture.

The peculiarity of our case underscores the need for additional diagnostic markers to aid in the diagnosis of biliary stricture, as it encompasses a broad range of differential diagnoses, including both malignant and benign conditions. When all investigations fail to yield a definitive diagnosis, the need for specialized testing, such as a liver biopsy or serum markers, can play a crucial role in aiding or supporting the diagnosis.

## Conclusions

In conclusion, the current study reported that our patient did not match all the criteria of IgG4-related sclerosing cholangitis. However, the patient was started on corticosteroid therapy given the elevated serum IgG4 and would require serial ERCP for dilatation of the biliary stricture. Thus, this study highlights the heterogeneity of the IgG4-related disease spectrum.
